# A 3-DNA methylation signature as a novel prognostic biomarker in patients with sarcoma by bioinformatics analysis

**DOI:** 10.1097/MD.0000000000026040

**Published:** 2021-05-21

**Authors:** Xiao-Wei Wang, Qi Sun, Shi-Bin Xu, Chao Xu, Chen-Jie Xia, Qi-Ming Zhao, Hua-Hui Zhang, Wei-Qiang Tan, Lei Zhang, Shu-Dong Yao

**Affiliations:** a Department of Plastic Surgery, Zhejiang Hospital; b Department of Orthopedic Surgery, Fuyang Orthopedics and Traumatology Hospital, Zhejiang Chinese Medical University; c Department of Orthopedic Surgery, The First People's Hospital of Xiaoshan District; d Department of Oncology, Zhejiang Cancer Hospital; e Department of Orthopedic Surgery, Li Hui-Li Hospital, Ningbo; f Department of Plastic Surgery, Sir Run Run Shaw Hospital, Zhejiang University College of Medicine; g Department of Orthopedic Surgery, Xiaoshan Hospital of Traditional Chinese Medicine, Zhejiang Chinese Medical University, Hangzhou; h Department of Nephrology, Huzhou Hospital of Traditional Chinese Medicine, Zhejiang Chinese Medical University, Huzhou, Zhejiang, China.

**Keywords:** DNA methylation, prognosis model, sarcoma, signature, The Cancer Genome Atlas

## Abstract

**Background:**

Tumor-specific DNA methylation can potentially be a useful indicator in cancer diagnostics and monitoring. Sarcomas comprise a heterogeneous group of mesenchymal neoplasms which cause life-threatening tumors occurring throughout the body. Therefore, potential molecular detection and prognostic evaluation is very important for early diagnosis and treatment.

**Methods:**

We performed a retrospective study analyzing DNA methylation of 261 patients with sarcoma from The Cancer Genome Atlas (TCGA) database. Cox regression analyses were conducted to identify a signature associated with the overall survival (OS) of patients with sarcoma, which was validated in a validation dataset.

**Results:**

Three DNA methylation signatures were identified to be significantly associated with OS. Kaplan–Meier analysis showed that the 3-DNA methylation signature could significantly distinguish the high- and low-risk patients in both training (first two-thirds) and validation datasets (remaining one-third). Receiver operating characteristic (ROC) analysis confirmed that the 3-DNA methylation signature exhibited high sensitivity and specificity in predicting OS of patients. Also, the Kaplan–Meier analysis and the area under curve (AUC) values indicated that the 3-DNA methylation signature was independent of clinical characteristics, including age at diagnosis, sex, anatomic location, tumor residual classification, and histological subtypes.

**Conclusions:**

The current study showed that the 3-DNA methylation model could efficiently function as a novel and independent prognostic biomarker and therapeutic target for patients with sarcoma.

## 1 Introduction

Sarcomas are a diverse group of mesodermal malignancies occurring at all ages, and are relatively rare, accounting for <1% of all adult cancers in the United States.^[1]^ These malignancies can arise from virtually any location throughout the body and comprise >50 histological subtypes.^[2]^ According to the type of tissue of primary manifestation, sarcomas can be grouped into 2 generalized groups: soft tissue sarcoma (liposarcoma, fibrosarcoma, undifferentiated pleomorphic sarcoma, leiomyosarcoma, and rhabdomyosarcoma) and bone sarcoma (osteosarcoma and chondrosarcoma).^[3]^ This histological heterogeneity makes sarcomas extremely difficult to accurately diagnose and treat. Therefore, they are quite deadly due to frequently delayed diagnosis and advanced disease at presentation. Assessment of patients prior to therapy may aid in forming a risk-adapted approach and guide the development of future personalized treatment strategies. Molecular biomarkers have been proven to be of great prognostic value for tumors, as they can provide more information and insight into the mechanisms of tumorigenesis.^[4]^ Consequently, it is urgent to identify effective prognostic biomarkers for accurate prognosis and targeted therapy in sarcoma patients.

DNA methylation is an epigenetic modification that is closely connected with gene expression regulation,^[5]^ and its signatures have great potential to become routine clinical cancer biomarkers due to their sensitivity, specificity, and ease of analysis.^[6]^ The methylation at particular subsets of CpG islands has been the main focus for research in recent years. DNA methylation is highly concentrated in the CpG islands within the promoter region of genes, and is strongly related to the silence of tumor suppressor genes and subsequent oncogenesis.^[7]^ Moreover, epigenetic alterations, such as aberrant DNA methylation have great utility for cancer diagnosis in the early stage due to several advantages over other molecular markers, including their appearance early in tumorigenesis^[7,8]^; wide distribution in the tumor tissue^[6]^; and consistency across a larger genomic region, so that multiple CpG dinucleotides can be used for detection.^[9]^ Therefore, tumor methylation research offers eminently practical perspectives for revealing potential diagnostic biomarkers in order to improve the survival rate. There have been numerous studies recently on DNA methylation as a biomarker for diagnosis and treatment guidance for some sarcoma types.^[10–12]^ However, the relationship of DNA methylation with sarcoma patients prognosis has not been fully elucidated.

In the present study, we constructed, verified, and evaluated a novel 3-DNA methylation signature that effectively predicted cancer prognosis based on data of sarcoma patients derived from The Cancer Genome Atlas (TCGA) database. We explored the potential clinical significance of DNA methylation signatures serving as molecular prognostic biomarkers using the Kaplan–Meier method and receiver operating characteristic (ROC) analyses. Furthermore, we investigated the independence and reproducibility of identified DNA methylation biomarkers in different clinical subgroups.

## 2 Materials and methods

### 2.1 DNA methylation data from sarcoma tissues taken from TCGA dataset

We downloaded processed DNA methylation data based on Infinium Human Methylation 450 BeadChip (Illumina Inc., CA) and related clinical information on sarcoma patients from TCGA dataset (https://protal.gdc.cancer.gov/).^[13]^ Ethical approval was not necessary for this study because public datasets were analyzed. DNA methylation level was expressed as a ratio termed β value, measured in terms of methylated probe intensities relative to the sum of the methylated and unmethylated probe intensities for each CpG site. The standardized β values ranged from 0 (completely unmethylated) to 1 (completely methylated). Any sarcoma patients with missing clinical survival information were excluded from this study. The relationship between DNA methylation level at a particular CpG site and the patients’ corresponding survival of sarcoma was analyzed. Eventually, 261 samples with 374,796 DNA methylation sites were included for analysis. All included samples were randomly divided into 2 parts according to the DNA methylation series number: two-thirds were used as the training dataset for constructing the prognostic model, and one-third was used as the validation dataset to verify the accuracy of the model in predicting survival of sarcoma.

### 2.2 Statistical analyses

Overall survival (OS) was defined as the time from the date of a patient's first diagnosis to the date of sarcoma-related death or last follow-up. We first performed univariate Cox proportional hazard analysis and robustness analysis in the training dataset to screen methylation biomarkers that were significantly associated with OS of sarcoma as candidate biomarkers (*P* < .05). To increase the feasibility and reliability of clinical prognosis based on DNA methylation, we also performed robustness analysis to select these candidate biomarkers. Then, we used multivariate Cox stepwise regression analysis to further select the factors correlated with patient OS and constructed models comprising all combinations of factors that were screened from the candidate biomarkers as covariates. The model weighted by regression coefficients was defined as a risk score formula and was used to predict survival. The prognostic risk score for each patient was calculated according to this formula and these patients in the training group were classified into low- and high-risk groups using the median risk score as a demarcation point. To explore whether the hazard ratio (HR) was constant over time, we also verified the proportional hazards (PH) assumption.^[14]^ Subsequently, we used Kaplan–Meier curves with log-rank test to calculate the cumulative survival time and evaluate the differences in OS between high- and low-risk groups. Furthermore, we assessed the risk scores for utility in predicting patient OS using the area under the receiver operating characteristic (ROC) curve (AUC). The last step was to identify whether the DNA methylation signatures were an independent factor, by performing data stratified analysis. All statistical analyses were carried out using the R Program (version 3.6.1).

## 3 Results

### 3.1 Clinical characteristics of included patients

A total of 261 patients clinically and pathologically diagnosed with sarcoma were included in this study. Among these patients, there were 119 men (45.60%) and 142 women (54.41%). The age of these patients ranged from 20 to 90 years with a median age of 61 years, and median overall survival (OS) was 550 days. The tumor histologic classification was assigned according to the type of tissue of primary manifestation. In the present study, we divided the histologic type of sarcomas into the following categories: dedifferentiated liposarcoma, leiomyosarcoma, myxofibrosarcoma, undifferentiated pleomorphic sarcoma (UPS), pleomorphic malignant fibrous histiocytoma (MFH)/undifferentiated pleomorphic sarcoma, giant cell MFH/undifferentiated pleomorphic sarcoma with giant cells, synovial sarcoma, malignant peripheral nerve sheath tumor (MPNST), and desmoid tumor. Anatomic sites were varied and included upper extremity, lower extremity, upper abdomen, lower abdomen, chest, head and neck, ovary, uterus, and superficial trunk. Tumor residual disease was classified into RX, R0, R1, and R2 according to American Joint Committee on Cancer (AJCC) R classification. The clinical characteristics of all patients are summarized in Table 1.

**Table 1:** Clinicopathological characteristics of sarcoma patients from TCGA database.

		Total (N = 261)		Training dataset (N = 157)		Validation dataset (N = 104)	
Characteristics	Groups	No.	%	No.	%	No.	%
Gender	Male	119	45.60	70	44.59	49	47.12
	Female	142	54.41	87	55.41	55	52.88
Age at diagnosis	Median	61		61		60	
	Range	20–90	20–90	24–90			
	>55	173	66.28	104	66.24	69	65.71
	≤55	88	33.72	53	33.76	36	34.29
Subtypes	Dedifferentiated liposarcoma	59	22.61	38	24.20	21	20.19
	Leiomyosarcoma	105	40.23	68	43.31	37	35.58
	Myxofibrosarcoma	25	9.58	15	9.55	10	9.62
	UPS	21	8.05	10	6.37	11	10.58
	MFH	29	11.11	15	9.55	14	13.46
	Giant cell MFH	1	0.38	0	0	1	0.96
	Synovial sarcoma	10	3.83	6	3.82	4	3.85
	MPNST	9	3.45	5	3.18	4	3.85
	Desmoid tumor	2	0.77	0	0	2	1.92
Anatomic location^∗^	Upper extremity	12	4.62	7	4.49	5	4.81
	Lower extremity	73	28.08	41	26.28	32	30.77
	Upper abdomen	99	38.08	59	37.82	40	38.46
	Lower abdomen	16	6.15	8	5.13	8	7.69
	Chest	13	5.00	8	5.13	5	4.81
	Head and neck	5	1.92	4	2.56	1	0.96
	Ovary	1	0.38	1	0.64	0	0
	Uterus	29	11.15	19	12.18	10	9.62
	Superficial trunk	12	4.62	9	5.77	3	2.88
Tumor residual^∗^	RX	26	10.00	17	10.90	9	8.65
	R0	155	59.62	89	57.05	66	63.46
	R1	70	26.92	44	28.21	26	25.00
	R2	9	3.46	6	3.85	3	2.88
Vital status	Alive	185	70.88	110	70.06	75	72.12
	Dead	76	29.12	47	29.94	29	27.88

### 3.2 Identification of prognostic DNA methylation markers in the training dataset

To explore the clinical role of DNA methylation biomarkers in sarcoma patient prognosis, we first identified 35,499 DNA methylation sites that were significantly (*P* < .05) associated with the OS of sarcoma patients to serve as candidate biomarkers using univariate Cox proportional hazard regression analysis. Moreover, 16 DNA methylation sites were selected from these candidate biomarkers after robustness analysis (Table 2).

**Table 2:** Top 16 DNA methylation sites significantly associated with the OS of sarcoma patients in the training dataset.

Probe ID	Hazard. ratio	95% CI	*P* value
cg00187535	1.03	1.01–1.04	.001379
cg07814289	1.03	1.02–1.05	.000165
cg08462924	1.04	1.02–1.06	.000472
cg08473330	1.02	1.01–1.03	.000283
cg09347923	1.08	1.03–1.13	.000718
cg09494609	1.03	1.02–1.04	.000001
cg09501372	1.05	1.03–1.07	.000035
cg09588555	1.05	1.03–1.08	.000006
cg14144025	1.03	1.01–1.04	.000079
cg15963326	1.02	1.01–1.03	.000057
cg16316162	1.03	1.02–1.04	.000001
cg19340420	1.02	1.01–1.03	.000012
cg19357499	1.04	1.03–1.06	.000001
cg24738592	1.04	1.03–1.05	.000001
cg24937735	0.97	0.96–0.99	.000007
cg25958857	1.03	1.02–1.04	.000001

**Figure 1:**
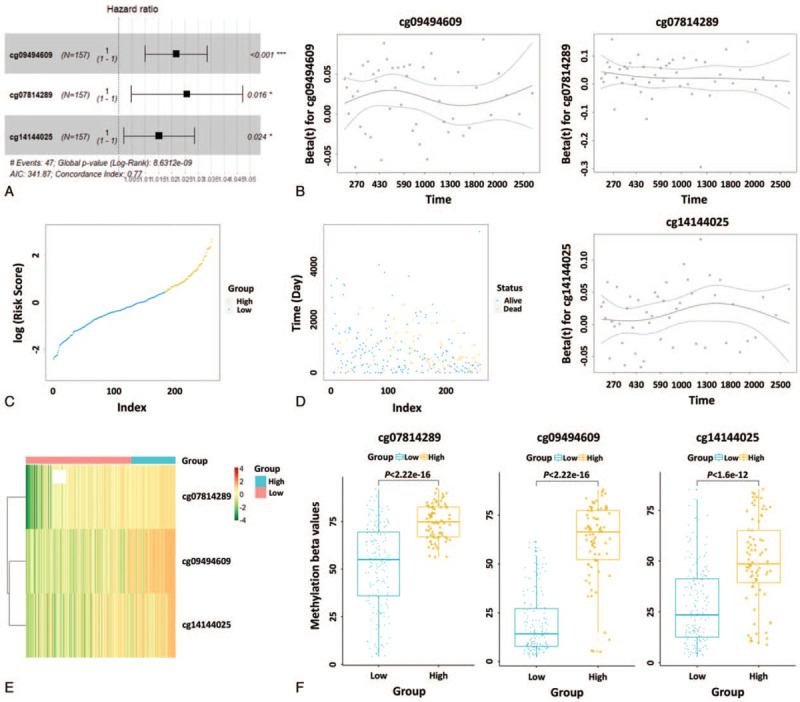
Risk score analysis of the 3-DNA methylation signature of sarcoma. (A) Forest plots of the 3-DNA methylation signature; all 3 methylation sites had positive coefficients. (B) The test of proportional hazards (PH) assumption based on Schoenfeld residuals; the residuals of the 3-DNA methylation signature are time-independent. (C) Distribution of high and low risk scores of 3 DNA methylation sites over entire TCGA dataset (N = 261). (D) Survival time and status of patients based on the high and low risk scores of 3 DNA methylation sites over entire TCGA dataset (N = 261). (E) Heatmap of the 3-DNA methylation signature in sarcoma patients. Each column represents a patient and each row a DNA methylation site. The methylation levels of the 3 sites are displayed in different colors. From green to red, expression gradually increases. (F) Boxplots of methylation β values in samples of patients in high- and low-risk groups in the training dataset. TCGA = The Cancer Genome Atlas.

Next, we performed multivariate Cox stepwise regression analysis and 3 methylation sites (cg07814289, cg09494609, and cg14144025) were ultimately screened as the optimum prognostic model for predicting the OS of patients with sarcoma (Table 3). As shown in Fig. 1A, all 3 methylation sites had positive coefficients, indicating a correlation between hypermethylation levels and short OS. We were thus able to establish a risk scoring formula for predicting OS based on the DNA methylation levels and regression coefficients of 3 methylation site results, as follows: Risk score = 0.025 × β value of cg07814289 + 0.021 × β value of cg09494609 + 0.015 × β value of cg14144025. Importantly, the 3-DNA methylation signature (cg07814289: *P* = .64, cg09494609: *P* = .87, cg14144025: *P* = .34) showed agreement with the proportional hazards (PH) assumption (Fig. 1B). All patient data from TCGA was divided into low-risk and high-risk groups according to the median of the risk score (Fig. 1C).

**Table 3:** Three significantly survival-related methylation sites in the training dataset.

Probe ID	Chromosomal location	Gene symbol	CGI coordinate	Feature type	*P* value^∗^	Coef.^†^	*P* value^†^
cg07814289	chr9:128218781-128218782	*DNM1*	chr9:128218621-128219038	Island	.000165	0.025	.015839
cg09494609	chr12:53054396-53054397	*RP11-983P16.4*	chr12:53054224-53054622	Island	.000001	0.021	.000257
cg14144025	chr4:13535009-13535010	*LINC01097*	chr4:13536022-13536349	N_Shore	.000079	0.015	.023773

Furthermore, we observed the distribution of all patients’ status, and the results showed that there were many more deaths in the high-risk group than in the low-risk group (Fig. 1D). As shown in the heatmap, the 3 DNA methylation levels were up-regulated with increasing risk score (Fig. 1E). Meanwhile, for these 3 DNA methylation sites, high-risk patients exhibited significantly higher methylation levels (Fig. 1F) (*P* < .01, Mann–Whitney *U* test).

### 3.3 Association between 3-DNA methylation signature and OS of patients in the training and validation datasets

**Figure 2:**
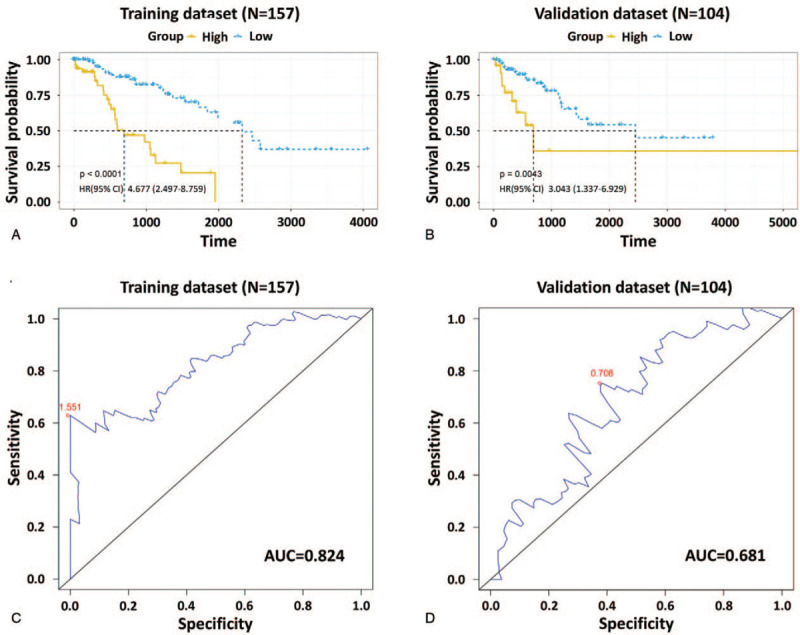
Kaplan–Meier and ROC analyses of the 3-DNA methylation signature in predicting the OS of patients with sarcoma. Kaplan–Meier estimates of the OS for high- and low-risk patient cohorts grouped by the 3-DNA methylation signature in the training dataset (N = 157) (A) and the validation dataset (N = 104) (B). (C) ROC analysis of sensitivity and specificity of the 3-DNA methylation signature in predicting patients’ OS in the training dataset, with an AUC of 0.824. (D) ROC analysis in the validation dataset, with an AUC of 0.681. AUC = area under curve, OS = overall survival, ROC = receiver operating characteristic.

According to the results of multivariate Cox regression analysis, the 3-DNA methylation signature was significantly associated with the OS of patients (Table 2). We performed Kaplan–Meier analysis in both the training and validation datasets to determine the potential predictive value of this 3-DNA methylation signature for the prognosis of sarcoma. As expected, the survival of patients in the high-risk group was significantly (*P* < .0001, HR: 4.677, 95% confidence interval [CI] of HR: 2.497–8.759) worse in comparison with patients in the low-risk group (Fig. 2A). This was also confirmed in the validation dataset (*P* = .0043, HR: 3.043, 95% CI of HR: 1.337–6.929) (Fig. 2B). These results indicated that the 3-DNA methylation signature could effectively stratify patients into high- and low-risk groups, implying its significance for prediction of prognosis.

To evaluate the sensitivity and specificity of the 3-DNA methylation signature in predicting survival, we calculated the AUC values of the ROC curves through ROC analysis in both datasets. The AUC of the 3-DNA methylation signature was 0.824 and 0.681 in the training and validation datasets, respectively (Fig. 2C and D). These results indicated that the 3-DNA methylation signature had high sensitivity and specificity as well as good discriminatory capacity for predicting OS of patients with sarcoma.

### 3.4 Independent prognostic ability of DNA methylation signature in OS prediction, considering other clinical factors

**Figure 3:**
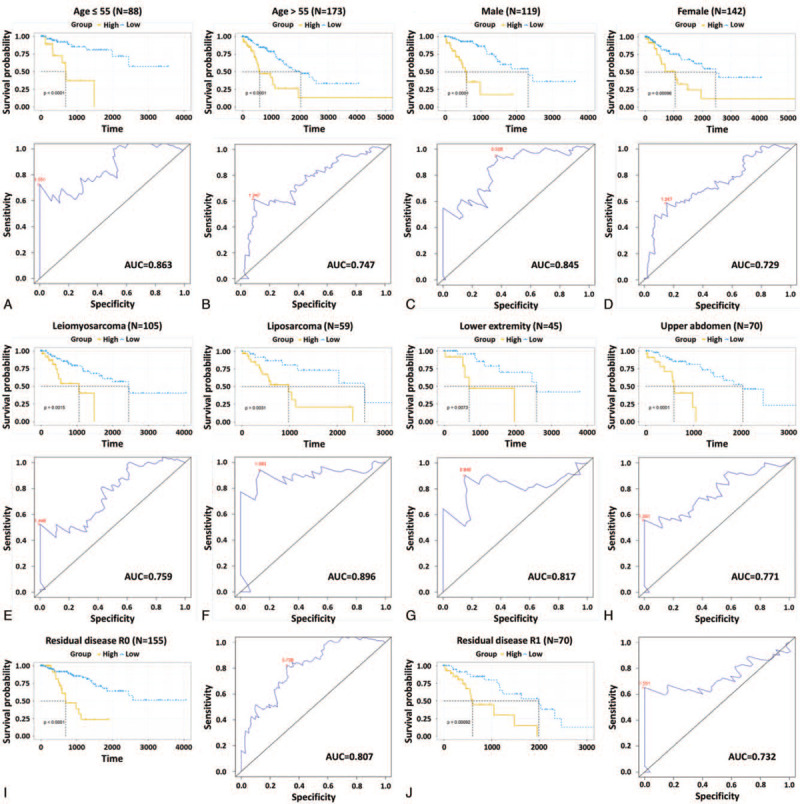
Kaplan–Meier and ROC analyses of sarcoma patients with different ages (A and B), genders (C and D), histological subtypes (E and F), anatomic locations (G and H), and residual disease R classifications (I and J). Kaplan–Meier estimates of the patients’ OS and ROC curves show the sensitivity and specificity of the 3-DNA methylation signature in predicting the patients’ OS. OS = overall survival, ROC = receiver operating characteristic.

We then wanted to know whether the 3-DNA methylation signature was an independent predictor for patients with sarcoma. Clinical and pathological characteristics, such as age, sex, histological type, anatomic location, and tumor residual have been considered predominant predictors for determining prognosis of sarcoma. Age is an important determinant of sarcoma occurrence. The mean age at diagnosis for soft tissue sarcoma and malignant bone tumors was 58 and 40 years of age, respectively, according to the data provided by the onal Center for Health Statistics (NCHS) and surveillance, epidemiology, and end results (SEER).^[2]^ All patients were divided into 2 groups based on age at initial diagnosis: ≤55 (N = 88, 33.72%) and >55 (N = 173, 66.28%). Kaplan–Meier curves showed that patients in the high-risk group had significantly (*P* < .01) shorter OS, and the AUC values were 0.863 and 0.747 respectively for the 2 age cohorts (Fig. 3A and B), suggesting that the 3-DNA methylation was independent of age. Meanwhile, previous research has shown that female hormones have a potential role in sarcoma development.^[15]^ Irrespective of sex, the patients in the low-risk group had significantly (*P* < .01) longer OS compared with patients in the high-risk group, and the AUC values were 0.845 and 0.729, in both male (N = 119, 45.6%) and female (N = 142, 54.41%) cohorts (Fig. 3C and D). As for the histological subtypes, taking into account the number of samples, we verified the predictive performance of the 3-DNA methylation signature in dedifferentiated liposarcoma (N = 59) and leiomyosarcoma (N = 105). The difference (*P* < .01) in the OS between the 2 groups was also observed, and the AUC values were 0.896 and 0.759, respectively (Fig. 3E and F). The lower extremity (Thigh/knee, N = 45) and upper abdomen (retroperitoneum, N = 70) subgroups were also included for these analyses due to small numbers in the other subgroups. Kaplan–Meier and ROC analyses demonstrated that the OS of patients in the low-risk group was much improved (*P* < .01) in comparison with that of patients in the high-risk group (Fig. 3G and H). Recent research has highlighted the fact that the presence of residual disease is an adverse prognostic factor.^[16]^ The present data showed that the 3-DNA methylation signature could provide a good reference for different residual disease groups (R0 and R1) owing to the effectiveness of risk stratification (Fig. 3I and J). All these results indicated that the 3-DNA methylation signature was an independent prognostic predictor for sarcoma patients.

## 4 Discussion

Sarcomas have considerable heterogeneity with respect to age of onset, anatomic location, and cells of mesenchymal origin. Because of this, sarcomas are particularly difficult to diagnose, leading to debate surrounding the sufficiency of histological diagnosis versus the need for ancillary molecular diagnostics.^[17]^ Tumor cells have a fundamentally different DNA methylation profile from normal original cells.^[5]^ Some of these differences do not occur in any normal cell types and are tumor-specific.^[18]^ In recent years, the importance of DNA methylation in the development of sarcoma has been increasingly acknowledged. Previous studies have demonstrated that DNA methylation signatures are able to reliably assign bone sarcomas to osteosarcoma, Ewing sarcoma, and synovial sarcoma, thereby providing a DNA-methylation-based classifier.^[12]^ Thus, the concept of detecting epigenetic alterations is transforming into clinical reality. TCGA database provides a large quantity of samples with a variety of clinical characteristics. Based on a TCGA dataset that included 261 sarcoma samples, the current study identified a prognostic signature which contained 3 methylation sites (cg07814289, cg09494609, and cg14144025) and corresponded to 3 genes (*DNM1*, *RP11-983P16.4*, and *LINC01097*) by combining differential methylation analysis, survival analysis, ROC analysis, and Cox regression analysis.

Interestingly, previous studies have shown that these 3 genes are associated with cancers. *DNM1* (Dynamin 1) is located on chromosome 9q34.11 and encodes DNM1 that is a GTPase involved in synaptic vesicle fission for receptor-mediated endocytosis on the presynaptic plasma membrane.^[19]^ DNM1 was discovered to be the critical protein responsible for regulating balance fusion and fission events of mitochondria in order to adapt mitochondrial morphology to altered physiological needs. DNM1 binds to the mitochondrial outer membrane via Fis1 and Mdv1 and assembles into higher oligomers at the mitochondrial surface, promoting the formation of rings and spirals that divide the organelle in a GTP-dependent manner.^[20]^ Studies show that DNM1 is a hub gene in various tumor tissues such as pediatric medulloblastoma^[21]^ and glioblastoma Multiforme.^[22]^*RP11-983P16.4* is a long non-coding RNA (lncRNA) which is located on chromosome 12. In a previous study, *RP11-983P16.4* was found to be significantly correlated with patients’ metastasis-free survival and it can be a useful prognostic marker to predict metastatic risk in breast cancer patients.^[23]^*LINC01097* is also a lncRNA that is located on chromosome 4. In human breast cancer MCF-7 cells, *LINC01097* is highly differentially upregulated and is associated with ribonucleoprotein (RNP) complex, which plays a significant role in pre-mRNA processing, mRNA stability, and translation mechanisms.^[24]^ Although the functional mechanism of these 3 genes still needs further elucidation, their methylation has prominent correlations with the prognosis of patients with sarcoma and may serve as an effective potential diagnostic marker and therapeutic target for sarcoma.

In the present study, our prognostic model based on 3 key DNA methylation signatures was able to stratify patients with sarcoma into high- and low-risk groups which exhibited significant differences in terms of survival. The accuracy of the prognostic model was validated by the validation dataset. Given the molecular and genetic heterogeneity of sarcoma, we subsequently analyzed whether the prognostic ability of the 3-DNA methylation signature was independent of clinical characteristics. Some factors may affect the independence of our prognostic model. Sarcomas, especially soft tissue sarcomas, osteosarcoma, and Ewing sarcoma, occur more frequently in young adults and adolescents compared with other cancers.^[2]^ The location of the primary tumor has been discovered to be one of the most important prognostic variables for soft tissue sarcomas in a previous study.^[25]^ One case-control study in Northern Italy investigated the potential association across a wide array of female-hormone-related factors, and indicate that women who become pregnant with their first child at later ages (>29 years old) are at high risk for sarcomas.^[15]^ The tumor residual R classification describes the tumor status following treatment and denotes absence or presence of residual tumor after treatment; this reflects the effects of therapy, influences further therapeutic procedures, and is a strong predictor of prognosis.^[26]^ Kaplan–Meier analysis and AUC values were thus used to assess the age at diagnosis, sex, anatomic location, tumor residual classification, and histological subtype independence of the 3-DNA methylation signature in predicting patients OS. The results show that the 3-DNA methylation signature exhibits prognostic power for all subgroups (in which patients with sarcoma can be further classified into high-risk and low-risk groups with significantly different OS prospects) indicating that the 3-DNA methylation signature is independent of clinical characteristics, including age at diagnosis, sex, anatomic location, tumor residual classification, and histological subtypes.

However, there are also some limitations in this study. First, we lack information on the mechanisms behind the prognostic ability of these 3 methylation genes in sarcoma, and additional experimental research on these genes should provide important data to further enhance our understanding of their functional roles. Second, some subgroups were not included for independent analysis due to small sample size, and the independence of these subgroups needs further research. Finally, although we validated our prognostic model with the validation dataset, the signature has not been tested prospectively in a clinical trial.

In conclusion, using genome-wide analysis of DNA methylation data of 261 patients, this study shows that a 3-DNA methylation signature is prominently associated with the OS of patients with sarcoma. The 3-DNA methylation signature is not only independent of clinical characteristics including age at diagnosis, sex, anatomic location, tumor residual classification, and histological subtypes, but also exhibits good ability in predicting OS of patients. Therefore, the 3-DNA methylation signature may serve as a novel independent prognostic biomarker to predict the OS of patients with sarcoma.

## Author contributions

**Data curation:** Qi Sun.

**Formal analysis:** Xiao-Wei Wang.

**Funding acquisition:** Hua-Hui Zhang.

**Software:** Xiao-Wei Wang, Chao Xu.

**Validation:** Chen-Jie Xia.

**Visualization:** Shi-Bin Xu.

**Writing – original draft:** Lei Zhang.

**Writing – review & editing:** Qi-Ming Zhao, Wei-Qiang Tan, Shu-Dong Yao.

## References

[1] Siegel RL Miller KD Jemal A. Cancer statistics, 2019. CA Cancer J Clin 2019;69:07–34. 10.3322/caac.21551 30620402

[2] Burningham Z Hashibe M Spector L Schiffman JD. The epidemiology of sarcoma. Clin Sarcoma Res 2012;2:14. 23036164 10.1186/2045-3329-2-14 PMC3564705

[3] Skubitz KM D’Adamo DR. Sarcoma. Mayo Clin Proc 2007;82:1409–32. 17976362 10.4065/82.11.1409

[4] Ransohoff DF. Cancer. Developing molecular biomarkers for cancer. Science (New York, NY) 2003;299:1679–80. 10.1126/science.1083158 12637728

[5] Kulis M Esteller M. DNA methylation and cancer. Adv Genet 2010;70:27–56. 20920744 10.1016/B978-0-12-380866-0.60002-2

[6] Roy D Tiirikainen M. Diagnostic power of DNA methylation classifiers for early detection of cancer. Trends Cancer 2020;6:78–81. 32061307 10.1016/j.trecan.2019.12.006 PMC7188195

[7] Laird PW. The power and the promise of DNA methylation markers. Nat Rev Cancer 2003;3:253–66. 12671664 10.1038/nrc1045

[8] Kanwal R Gupta K Gupta S. Cancer epigenetics: an introduction. Methods Mol Biol 2015;1238:03–25. 10.1007/978-1-4939-1804-1_1 25421652

[9] Hao X Luo H Krawczyk M. DNA methylation markers for diagnosis and prognosis of common cancers. Proc Natl Acad Sci U S A 2017;114:7414–9. 28652331 10.1073/pnas.1703577114 PMC5514741

[10] Tombolan L Poli E Martini P. Global DNA methylation profiling uncovers distinct methylation patterns of protocadherin alpha4 in metastatic and non-metastatic rhabdomyosarcoma. BMC Cancer 2016;16:886. 27842508 10.1186/s12885-016-2936-3 PMC5109816

[11] Li J Xing X Li D. Whole-Genome DNA methylation profiling identifies epigenetic signatures of uterine carcinosarcoma. Neoplasia 2017;19:100–11. 28088687 10.1016/j.neo.2016.12.009 PMC5237802

[12] Wu SP Cooper BT Bu F. DNA methylation-based classifier for accurate molecular diagnosis of bone sarcomas. JCO Precis Oncol 2017;2017: PO.17.00031. 10.1200/PO.17.00031 PMC5772901 29354796

[13] Hudson TJ Anderson W Artez A. International network of cancer genome projects. Nature 2010;464:993–8. 20393554 10.1038/nature08987 PMC2902243

[14] Uno H Claggett B Tian L. Moving beyond the hazard ratio in quantifying the between-group difference in survival analysis. J Clin Oncol 2014;32:2380–5. 24982461 10.1200/JCO.2014.55.2208 PMC4105489

[15] Fioretti F Tavani A Gallus S Negri E Franceschi S La Vecchia C. Menstrual and reproductive factors and risk of soft tissue sarcomas. Cancer 2000;88:786–9. 10679647 10.1002/(sici)1097-0142(20000215)88:4<786::aid-cncr8>3.0.co;2-m

[16] Grimer R Parry M James S. Inadvertent excision of malignant soft tissue tumours. EFORT Open Rev 2019;4:321–9. 31312520 10.1302/2058-5241.4.180060 PMC6598609

[17] Italiano A Di Mauro I Rapp J. Clinical effect of molecular methods in sarcoma diagnosis (GENSARC): a prospective, multicentre, observational study. Lancet Oncol 2016;17:532–8. 26970672 10.1016/S1470-2045(15)00583-5

[18] Wan J Massie C Garcia-Corbacho J. Liquid biopsies come of age: towards implementation of circulating tumour DNA. Nat Rev Cancer 2017;17:223–38. 28233803 10.1038/nrc.2017.7

[19] Ferguson SM De Camilli P. Dynamin a membrane-remodelling GTPase. Nat Rev Mol Cell Biol 2012;13:75–88. 22233676 10.1038/nrm3266 PMC3519936

[20] Müller M Lu K Reichert AS. Mitophagy and mitochondrial dynamics in Saccharomyces cerevisiae. Biochim Biophys Acta 2015;1853:2766–74. 25753536 10.1016/j.bbamcr.2015.02.024

[21] Huang P Guo YD Zhang HW. Identification of hub genes in pediatric medulloblastoma by multiple-microarray analysis. J Mol Neurosci 2020;70:522–31. 31820345 10.1007/s12031-019-01451-4

[22] Patel VN Gokulrangan G Chowdhury SA. Network signatures of survival in glioblastoma multiforme. PLoS Comput Biol 2013;9:e1003237. 24068912 10.1371/journal.pcbi.1003237 PMC3777929

[23] Sun J Chen X Wang Z. A potential prognostic long non-coding RNA signature to predict metastasis-free survival of breast cancer patients. Sci Rep 2015;5:16553. 26549855 10.1038/srep16553 PMC4637883

[24] Tripathi R. Extricating Novel lncRNAs Regulatory Expression Profiling in Breast Cancer. International Symposium of Materials on Regenerative Medicine (2017 ISOMRM) 2017;Chung Yuan Christian University, Taiwan

[25] Pisters PW Leung DH Woodruff J. Analysis of prognostic factors in 1,041 patients with localized soft tissue sarcomas of the extremities. J Clin Oncol 1996;14:1679–89. 8622088 10.1200/JCO.1996.14.5.1679

[26] Hermanek P Wittekind C. Residual tumor (R) classification and prognosis. Semin Surg Oncol 1994;10:12–20. 8115781 10.1002/ssu.2980100105

